# Screening of pregnant women attending the antenatal care clinic of a tertiary hospital in eastern Saudi Arabia for *Chlamydia trachomatis* and *Neisseria gonorrhoeae* infections

**DOI:** 10.4103/2589-0557.74976

**Published:** 2010

**Authors:** Alhusain J. Alzahrani, Obeid E. Obeid, Manal I. Hassan, Abdalaziz A. Almulhim

**Affiliations:** Department of Microbiology, College of Medicine, Dammam University, Kingdom of Saudi Arabia; 1 Department of Obstetrics & Gynecology, College of Medicine, Dammam University, Kingdom of Saudi Arabia

**Keywords:** *Chlamydia trachomatis*, *Neisseria gonorrhoeae*, sexually transmitted infections

## Abstract

**Inroduction:**

Of the “top ten” sexually transmitted infections, *Chlamydia trachomatis* and *Neisseria gonorrhoeae* are ranked second and fifth, respectively, worldwide.

**Aim:**

The aim of this study was to screen the pregnant women for *C. trachomatis* and *N. gonorrhoeae* infections and to detect antimicrobial resistance pattern of *N. gonorrhoeae*.

**Materials and Methods:**

This study was a prospective, hospital-based analysis of a random sample of pregnant women visiting the antenatal clinic of a tertiary hospital in eastern Saudi Arabia. Endocervical and high vaginal swabs were collected both from pregnant women and female patients attending gynecology clinic with lower genital tract infection (control group). *C. trachomatis* antigen was detected using enzyme-linked immunosorbent assay (ELISA). *N. gonorrhoeae* was detected by culture and identification of isolates, and antimicrobial susceptibility testing was performed. Statistical Package for Social Sciences (SPSS) version 13.0 and Chi-square test were used for statistical analysis.

**Results:**

*C. trachomatis* antigen was detected in 10.5% (10/95) and 34.4% (35/102) of pregnant women and control group, respectively (*P* < 0.001). The isolation rate of *N. gonorrhoeae* among pregnant women was 0.0% compared to 7.8% (8/102) among the control group (*P* < 0.01). *N. gonorrhoeae* were resistant to penicillin (62.5%), tetracycline (50%), ampicillin (25%), amoxycillin–clavulinic acid (25%) and ciprofloxacin (37.5%), while they were susceptible to cefepime, ceftriaxone, ceftazidime, spectinomycin, and cefuroxime.

**Conclusion:**

Screening of pregnant women for *C. trachomatis* infection should be included in the antenatal care in this area. The detection rate of both organisms among the control group highlights the importance of preventive strategies. Certain antibiotics previously used in treating gonorrhea are no longer effective.

## INTRODUCTION

*Chlamydia trachomatis* and *Neisseria gonorrhoeae* infections are associated with adverse pregnancy outcomes.[1–5] To the best of our knowledge, there are neither any published data on the prevalence of *C. trachomatis* or *N. gonorrhoeae* infections in eastern Saudi Arabia nor any information on the antimicrobial susceptibility pattern of *N. gonorrhoeae* in this area.

This study aimed at screening a random sample of pregnant women visiting the antenatal clinic of a tertiary hospital in eastern Saudi Arabia for *C. trachomatis* and *N. gonorrhoeae* infections and detection of antimicrobial resistance pattern of *N. gonorrhoeae*.

## MATERIALS AND METHODS

### Patients

The study population included a random sample of pregnant women visiting the antenatal care clinic of a tertiary hospital in eastern Saudi Arabia, who had no symptoms suggesting sexually transmitted diseases (STDs; vaginal discharge, dysuria, pelvic discomfort, pruritus, irritation) and who had no antibiotic treatment for 2 months before the date of examination. Female patients attending gynecology clinic of the same hospital with symptoms of lower genital tract infection were also included as matching controls. The study was taken over a period of 1 year (January 2005–February 2006). A written consent was obtained from each of the study participants.

For *C. trachomatis*, the exocervix was first wiped of exudate, a swab was inserted into the endocervical canal, passing the squamo-columnar junction, to permit the acquisition of columnar epithelial cells which are the main reservoir of the organism, firmly rotated and then withdrawn. Each swab was placed in the sample collection vial containing the buffer (1 ml) supplied with the commercially available enzyme-linked immunosorbent assay (ELISA) kit (Abbott’s Diagnostics, USA) to be stored immediately on arrival at −20° C, until tested. *Chlamydia* antigen was detected by ELISA according to the manufacturer’s instructions.

For *N. gonorrhoeae*, the exocervix was first wiped of exudate, a swab was then inserted and rotated 2 inches into the vagina, placed into the Stuart transport medium and sent to the laboratory. Cultures for *N. gonorrhoeae* were performed on chocolate agar and Thayer–Martin Medium and incubated in an enriched CO_2_ environment. After 24–48 hours, presumptive identification was made on the basis of colony morphology. Suspected colonies were Gram-stained and examined microscopically. Oxidase and catalase tests were performed. Colonies of Gram-negative diplococci positive for oxidase and catalase were further tested for carbohydrate fermentation with acid production.[6] Antimicrobial susceptibility testing was performed using the E test. Antimicrobial agents tested were, penicillin, ampicillin, amoxycillin–clavulinic acid, tetracycline, ceftriaxone, ceftazidime, cefepime, cefuroxime, ciprofloxacin and spectinomycin.

### Techniques used for data collection

An interview-based questionnaire for clinical and obstetric history was delivered. Data included age, nationality, religion, education level, occupation, history of previous abortion, gravidity, parity, use of contraceptives and symptoms of lower genital tract infection, if any.

### Statistical analysis

Data were analyzed using Statistical Package for Social Sciences (SPSS) version 13.0. Chi-square test was used to assess the association for categorical variables. In the case of sparse data, the Fisher’s exact probability was used as indicated. For continuous variables, independent *t*-test was used. Stepwise logistic regression was used to predict the outcome variable(s) from possible predictors. Level of significance was set to be <0.05 throughout the study.

## RESULTS

Endocervical and high vaginal swabs were collected both from a random sample of pregnant women, who had no symptoms suggesting STD and with no antibiotic treatment for 2 months before the date of examination, (test group, *n* = 95), and the female patients attending gynecology clinic with symptoms of lower genital tract infection (control group, *n* = 102). Most pregnant women declined to participate apparently to avoid the procedures particularly as they had no complaints. Additionally, some women were reluctant to undergo a pelvic examination, for personal or cultural reasons.

The distribution of pregnant women enrolled in the study according to age, nationality, religion, gravidity, parity and history of abortion is shown in Table 1.

**Table 1:** Distribution of pregnant women enrolled in the study according to age, nationality, religion, gravidity, parity and history of abortion

Characteristics	n = 95		
	No.		%
Age (years)			
<25	24		25.3
25-35	50		52.6
>35	21		22.1
Mean		29.9	
SD		7.0	
Nationality			
Saudi	71		74.7
Non-Saudi	24		25.3
Religion			
Muslim	90		94.7
Others	5		5.3
Gravidity			
1	22		23.2
2-4	38		40.0
5+	35		36.8
Parity			
0	25		26.3
1-4	48		50.5
5+	22		23.2
No. of abortions			
0	75		78.9
1 +	20		21.1

Of the 95 pregnant women tested, 10 (10.5%) were positive for *C. trachomatis* antigen. Table 2 shows that the mean age of positive cases was significantly higher than that of negative ones (34.2 versus 29.3 years, *P* = 0.038). A significantly higher detection rate of chlamydial infection was observed among multigravidae compared to primigravidae [13.7% (10/73) versus 0.0% (0/22), *P* = 0.009], among multipara compared to nullipara [12.9% (9/70) versus 4.0% (1/25), *P* = 0.013), as well as among women with history of abortion compared to those without such a history [25% (5/20) versus 6.7% (5/75), *P* = 0.032].

**Table 2:** Distribution of *C. trachomatis* positive and negative cases by age, nationality, religion, gravidity, parity and history of abortions

Characteristic	*C. trachomatis* +ve			*C. trachomatis* -ve			Test of significance
	No.		%	No.		%	
Age (years)							
<25	0		0.0	24		28.2	*t*-test = 2.11
25-35	6		60.0	44		51.8	*P* = 0.038
>35	4		40.0	17		20.0	
Mean		34.2			29.3		
SD		6.6			6.9		
Nationality							
Saudi	8		80.0	63		74.1	*χ*^2^= 0.16
Non-Saudi	2		20.0	22		25.9	*P* = 0.73
Religion							FEP = 0.56
Muslim	10		100.0	80		94.0	
Others	0		0.0	5		6.0	
Gravidity							
1	0		0.0	22		25.9	*χ*^2^ = 9.356
2-4	2		20.0	36		42.4	*P* = 0.009
5+	8		80.0	27		31.8	
Parity							
0	1		10.0	24		28.2	*χ*^2^ = 8.613
1-4	3		30.0	45		52.9	*P* = 0.013
5+	6		60.0	16		18.8	
No. of abortions							
0	5		50.0	70		82.4	*χ*^2^ = 5.635
1 +	5		50.0	15		17.6	*P* = 0.032

FEP = Fisher’s exact probability

Table 3 shows the results of the stepwise logistic regression analysis for determinants of *C. trachomatis* infection. Stepwise regression for four significant variables using univariate analysis (age, parity, gravity, abortion) resulted in a model that included gravidity only (χ^2^ = 7.99, *P* = 0.007). The odds ratio for gravidity is 1.37 [confidence interval (CI) = 1.09-1.72], meaning that the probability of getting the infection increases by 1.37 times with each increase by 1 in gravidity. Stepwise regression accounted for the high colinearity between parity and gravidity.

**Table 3:** Stepwise logistic regression analysis for factors affecting infection with *C. trachomatis*

Variable	Odds	Significance	95% CI for odds ratio		Model chi-square
			Lower	Upper
Variables in the equation					*χ*^2^ = 7.99 *P* = 0.007
Constant	0.026	<0.001			
Gravity	1.37	0.008	1.09	1.72	
Variables not in the equation					
Age		0.66			
Abortion		0.73			
Parity		0.73			

Regarding the control group, 35 out of 102 participants (34.4%) were positive for *C. trachomatis* antigen. Compared to the test group, where 10.5% detection rate was observed, the difference was found to be statistically significant (*P* < 0.001). *N. gonorrhoeae* was isolated from 8 out of 102 females of the control group (7.8%). Compared to a 0.0% isolation rate among the test group, the difference was found to be statistically significant (*P* < 0.01).

Regarding antimicrobial susceptibility testing of the eight *N. gonorrhoeae* isolates, antimicrobial resistance was detected to penicillin in 62.5% of isolates (95% CI: 28.95–96.05), tetracycline in 50% of isolates (95% CI: 15.35–84.65), ampicillin in 25% of isolates (95% CI: –5.01 to 55.01), amoxycillin–clavulinic acid in 25% of isolates (95% CI: –5.01 to 55.01) and ciprofloxacin in 37.5% of isolates (95% CI: 3.95–71.05). The organism was fully susceptible to cefepime, ceftriaxone, ceftazidime, spectinomycin, and cefuroxime.

## DISCUSSION

STDs are a major global cause of acute illness with severe medical and psychological consequences for millions of men, women and children. Despite the tracking difficulties, the estimated global annual incidence of curable sexually transmitted infections (STIs), excluding HIV and viral hepatitis, is 333 million cases, of which chlamydial infections represent 89 million cases while gonococcal infections represent 62 million cases.[7]

Information about STIs in Islamic countries is notably limited. Nongonococcal urethritis, trichomoniasis, and gonococcal urethritis are the most commonly reported STIs in Saudi Arabia.[8] The average annual incidence of nongonococcal urethritis and gonorrhea per 100,000 total population from 1995 to 1999 was 12.8 and 4.9, respectively.[8]

The prevalence of chlamydial genital infection in women varies in different groups and communities. The incidence of such infection in asymptomatic unselected pregnant women varies from 4 to 21%.[9] Only a few studies done in Saudi Arabia have been reported in the literature. In this study, 10.5% of randomly selected pregnant women were positive for *C. trachomatis* antigen. These results are more or less in keeping with other studies done in Saudi Arabia.[10,11] However, Ghazi *et al*.[12] reported 1.5% seroprevalence of *C. trachomatis* IgM among a group of randomly selected Saudi pregnant women. Considering IgM as an indicator for current infection, these results are in contrary to ours, which may be due to the difference in the detection technique used.

Screening of pregnant women was performed in different countries. The prevalence rates detected were 2.9% in north Ireland,[13] 9% in Zaire,[14] 14% in USA,[15] 4.8% in New Zealand,[16] and 4.2% in Japan.[17] These differences in the detection rates may be due to difference in cultures and social behaviors, and/or varying sensitivity of the detection techniques used, including the number of body sites sampled and the number of passages in case of diagnosis by tissue culture.

In this study, the mean age of positive cases was significantly higher than that of negative ones, being 34.2 versus 29.3 years. Similar finding was reported by Rastogi (25.8 versus 23.6 years).[18] However, the mean age of *Chlamydia* positive cases in the present study is higher than that reported in the literature,[13,16] where cervical infection is common in younger age groups. This is probably because in developed countries females are sexually active at a younger age.

In the present study, a significantly higher detection rate of chlamydial infection (13.7%) was observed among multigravidae compared to primigravidae (0.0% detection rate) as well as among multipara (12.9%) compared to nullipara (4.0%). The probability of getting the infection was found to be increased by 1.37 times with each increase by 1 in gravidity. Similar results were obtained from other studies which demonstrated a higher rate of infection among multigravidae compared to primigravidae (22.3% versus 20.3% and 23.8% versus 18.6%)[18,19] and among multipara compared to nullipara (0.92% versus 0.68%).[19] However, Shimano documented that the prevalence of *C. trachomatis* infection was significantly higher among women with no history of delivery.[17]

It has been documented that history of previous abortions suggests a higher risk for *C. trachomatis* infection.[20] Similarly, in the present study, 25% of women with a past history of abortion were positive for *C. trachomatis* antigen compared to 6.7% of those without such a history.

In this study, there were limitations to the interpretation of other risk factors such as socioeconomic status and previous STIs as no complete data could be retrieved.

With regard to the control group, 34.4% of females attending gynecology clinic were positive for *C. trachomatis* antigen. Similarly, Massoud *et al*.[21] detected anti chlamydial antibodies in the sera of 35% of female patients attending gynecological clinic, using the immunofluorescence test. On the other hand, El-Sheikh *et al*.[10] diagnosed *C. trachomatis* infection in the cervix uteri of 5.5% of gynecological patients and Jamjoom *et al*.[22] using culture, reported only 0.5% positivity. The contradiction between the results of these studies and our results may be due to the different techniques used and/or the different sample sizes.

The data on *N. gonorrhoeae* infection in Saudi pregnant women are very scanty. Also, there is no information on the antimicrobial susceptibility pattern of *N. gonorrhoeae* in the eastern Saudi Arabia. The prevalence of *N. gonorrhoeae* among pregnant women enrolled in this study was 0.0%. Negative results may be attributed to the very low prevalence of gonococcal infection in the country (average annual incidence of 4.9 per 100,000 population from 1995 to 1999).[8] The use of culture in this study, rather than molecular techniques, may be another contributing factor. Newer screening tests, including nucleic acid amplification and nucleic acid hybridization tests, have demonstrated sensitivity and specificity comparable to cervical culture.[23] However, the Center for Disease Control and Prevention has published recommendations that support the use of culture when screening for gonorrhea.[24] The sensitivity of culture varies widely, ranging from 61.8 to 92.6%, but remains high when the transport conditions are suitable.[23]

In this study, *N. gonorrhoeae* was detected in 7.8% of the control group, a high detection rate which cannot be explained, but it may be related to some unknown behavior of these patients. In a study conducted in Kuwait, it was reported that of all the patients who presented with signs and symptoms suggestive of STDs, seen over a 1-year period, diagnosis of gonorrhea was made in 31.5%, *Chlamydia* urethritis in 4.1%, concomitant gonorrhea and *Chlamydia* urethritis in 2.7%.[25]

The United States Preventive Services Task Force found insufficient evidence to recommend for or against routine screening for gonorrhea infection in pregnant women who are not at increased risk for infection.[23] On the other hand, screening of pregnant women at risk for gonorrhea is strongly recommended.[26]

Antibiotic resistance in *N. gonorrhoeae* has severely compromised successful treatment. In the current research, antimicrobial resistance of *N. gonorrhoeae* was detected to penicillin, tetracycline, ampicillin, amoxycillin–clavulinic acid and ciprofloxacin. The organism was fully susceptible to cefepime, ceftriaxone, ceftazidime, spectinomycin, and cefuroxime. These results are in keeping with those of other studies.[27–29]

As a result of the general misuse of antibiotics, there is continuing detection of bacterial isolates with decreased susceptibility to many of these antimicrobials. This issue remains a matter of interest and concern. In this study, because of the high prevalence of penicillin resistant strains, we recommend that all gonococcal isolates should be screened for beta lactamase production by a quick strip method;[30] all positive and doubtful results should then be confirmed by a plate or other methods. Additionally, there is an emergence of quinolone-resistant isolates (three out of eight isolates). On the other hand, the broad-spectrum cephalosporins (cefepime, ceftazidime, ceftriaxone, cefuroxime) demonstrated very high levels of activity (100% susceptibility) against *N. gonorrhoeae* isolates. Because of the above mentioned data, the current recommended gonorrhea treatment options are cephalosporins.

Local antimicrobial susceptibility monitoring is crucial to ensure appropriate treatment of gonorrhea. Standard regimens can be based on *in vitro* susceptibility determinations and should be modified when susceptibility patterns change.

Linking laboratory data to epidemiological data and identifying cases of resistance acquired both locally or abroad would permit a more complete picture of resistance by identifying further or more precisely groups at higher risk and thus allow health care practitioners to optimize the treatment of gonococcal infection.

Even though the findings of this study may not represent the pattern of resistance of *N. gonorrhoeae* all over the area, they, nevertheless, illustrate the need to establish an antimicrobial resistance surveillance system along with further studies on a larger sample size.

## CONCLUSION

Testing for *C. trachomatis* should be added to best-practice screening that is already carried out during pregnancy in this area. On the other hand, the results showed no evidence to recommend for screening pregnant women for gonorrhea, without being at risk. The detection rates of both the organisms among the control group highlights that appropriate preventive strategies are essential. The results also showed that certain antibiotics previously used in the treatment of gonorrhea are no longer effective.
